# Feasibility of nonspinal bony landmark-based image registration in CyberKnife ® robotic radiotherapy for pelvic and femoral bone tumors

**DOI:** 10.1093/jrr/rrag028

**Published:** 2026-04-24

**Authors:** Yasuhide Miyabe, Hiromu Yamanaka, Hiroto Seki, Yusuke Muroi, Shinri Nakano, Ryota Yamazaki, Azusa Awaya, Jyunetsu Mizoe

**Affiliations:** Sapporo High Functioning Radiotherapy Center, Sapporo Kojinkai Memorial Hospital, 1-16-1 Miyanosawa 2-jo, Nishi-ku, Sapporo-shi, Hokkaido 063-0052, Japan; Sapporo High Functioning Radiotherapy Center, Sapporo Kojinkai Memorial Hospital, 1-16-1 Miyanosawa 2-jo, Nishi-ku, Sapporo-shi, Hokkaido 063-0052, Japan; Sapporo High Functioning Radiotherapy Center, Sapporo Kojinkai Memorial Hospital, 1-16-1 Miyanosawa 2-jo, Nishi-ku, Sapporo-shi, Hokkaido 063-0052, Japan; Sapporo High Functioning Radiotherapy Center, Sapporo Kojinkai Memorial Hospital, 1-16-1 Miyanosawa 2-jo, Nishi-ku, Sapporo-shi, Hokkaido 063-0052, Japan; Sapporo High Functioning Radiotherapy Center, Sapporo Kojinkai Memorial Hospital, 1-16-1 Miyanosawa 2-jo, Nishi-ku, Sapporo-shi, Hokkaido 063-0052, Japan; Sapporo High Functioning Radiotherapy Center, Sapporo Kojinkai Memorial Hospital, 1-16-1 Miyanosawa 2-jo, Nishi-ku, Sapporo-shi, Hokkaido 063-0052, Japan; Sapporo High Functioning Radiotherapy Center, Sapporo Kojinkai Memorial Hospital, 1-16-1 Miyanosawa 2-jo, Nishi-ku, Sapporo-shi, Hokkaido 063-0052, Japan; Sapporo High Functioning Radiotherapy Center, Sapporo Kojinkai Memorial Hospital, 1-16-1 Miyanosawa 2-jo, Nishi-ku, Sapporo-shi, Hokkaido 063-0052, Japan

**Keywords:** CyberKnife, image registration, bone metastases, stereotactic radiotherapy

## Abstract

In stereotactic body radiotherapy for bone metastases using the CyberKnife system, spinal anatomy is generally employed as the alignment reference. However, when treating pelvic tumors such as those in the pubic bone or proximal femur, the spinal reference may be anatomically distant from the target, potentially compromising visualization and localization accuracy. This study evaluated the feasibility of using nonspinal bony landmarks, specifically the pubic bone and femoral neck, as reference points for image registration. Intrafractional positional deviations in six degrees of freedom and the frequency of intrafractional corrections were analyzed according to treatment site, including cases treated with conventional spine-based alignment. Alignment accuracy was visually and numerically verified by the treatment operator before beam delivery. Across all treatment sites, the mean translational and rotational deviations during treatment remained within ±0.5 mm and ± 0.5°, respectively. Furthermore, no substantial differences were observed in the mean frequency of intrafractional corrections among the different alignment approaches. These findings demonstrate that nonspinal tumor-adjacent bony landmarks can provide clinically acceptable accuracy comparable to conventional spine-based registration. To our knowledge, this is the first study to systematically assess the clinical feasibility of this approach in CyberKnife-based pelvic radiotherapy. The proposed method may serve as a practical and reliable alternative to spine-based alignment, particularly when the spine is remote from the target.

## INTRODUCTION

CyberKnife (Accuray Inc., Sunnyvale, CA, USA) is a stereotactic radiosurgery and stereotactic radiotherapy (SRT) system that achieves submillimeter accuracy through real-time image guidance. It utilizes a compact 6 MV X-band linear accelerator mounted on a highly flexible, multi-jointed robotic arm. This configuration facilitates precise beam delivery with six degrees of freedom (6DoF), enabling a wide range of beam angles and directions. The system can perform isocentric and nonisocentric irradiation, delivering high-energy photon beams from multiple trajectories to accurately target tumors while minimizing exposure to surrounding healthy tissues [1–3].

Among several image registration methods used in the system, the Xsight Spine Tracking System accurately tracks the position and orientation of the target using the spine as a landmark by registering the planning computed tomography (CT) images with X-ray images acquired during treatment. The X-ray images are obtained by irradiating two approximately orthogonal detectors using two diagnostic X-ray generators mounted on the ceiling of the treatment room. By comparing digitally reconstructed radiographs (DRRs) generated from the CT images with the actual X-ray images, subtle movements of bony structures are detected and corrected in real time throughout the treatment [4]. Furthermore, the system continuously acquires X-ray images during treatment and incorporates real-time corrections of the robotic arm based on image registration results. In a previous study, Fürweger *et al*. investigated the impact of intrafractional patient motion on target accuracy and reported that patient motion had minimal influence on the delivered dose distribution when image-guided beam alignment was employed [5].

Previous studies have reported that SRT using the CyberKnife system achieves favorable clinical outcomes within a limited number of treatment sessions for bone tumors [6, 7]. This treatment utilizes the aforementioned tracking system, where a vertebra located at the same level as the tumor is typically designated as the alignment center (imaging/mechanical center) for image registration.

Recently, clinical cases of CyberKnife treatment using the Xsight Spine Tracking System for tumors located near the pelvis have been reported, and Harada *et al*. demonstrated favorable local tumor control with minimal toxicity in SRT for cervical cancer [8].

However, when the sacrum, which is the vertebra closest to the pelvis, is used as the alignment center, the treatment region may lie outside or near the boundary of the X-ray imaging field in cases where the tumor is located far from the sacrum. As a result, verifying the target during irradiation becomes challenging.

Furthermore, such spatial discrepancies may amplify angular errors into significant positional deviations at the tumor site. For instance, an angular error of 1° at the alignment center corresponds to a vertical displacement of ~1.75 mm at a distance of 10 cm. Given that the CyberKnife system is characterized by high dose conformity and steep dose gradients in SRT, such geometric constraints could potentially compromise the accuracy of dose localization. Inoue *et al*. investigated irradiation errors caused by head rotation during treatment and reported that tumor displacement due to head rotation increases and significantly affects the dose distribution, when the distance from the isocenter to the tumor is large [9].

To address this issue, we proposed a novel image registration approach in which the irradiation site itself (e.g. the pubic bone or femoral neck) is designated as the alignment center.

To our knowledge, this is the first clinical study to evaluate the feasibility of nonspinal bony structures as alignment references in CyberKnife-based SRT. To evaluate the feasibility of the proposed method, we compared the results obtained using the proposed approach with those from the conventional spine-based tracking method to confirm that no substantial discrepancies were present.

## MATERIALS AND METHODS

Radiotherapy was performed using the CyberKnife M6 system (Accuray Inc., Sunnyvale, CA, USA), with radiation treatment planning conducted using the Multiplan version 5.3 (Accuray Inc., Sunnyvale, CA, USA). Planning CT images were acquired using a SOMATOM Perspective CT scanner (Siemens Healthineers, Forchheim, Germany).

This study was approved by the institutional review board (IRB No. 18000006, approved on 3 July 2025; Ref No. 2025-10) and conducted in accordance with the Declaration of Helsinki. Written informed consent was obtained from all participants.

A total of 24 patients who received treatment between April 2021 and January 2023 were included in this study. Thirteen patients (8 men and 5 women; median age, 65 years; range, 45–79 years) were treated using the new registration method. This group included five cases of right femoral tumors, three of left femoral tumors and five of pubic bone tumors. Based on histopathological and radiological findings, all lesions were identified as metastatic bone tumors. The comparison group comprised 11 patients (5 men and 6 women; median age, 63 years; range, 42–81 years) diagnosed with metastatic bone tumors of the thoracic or lumbar spine. In this group, the height of the metastatic lesion and the alignment center were matched, and treatment was performed using image registration with conventional spine alignment.

Patient immobilization was achieved using a Vac-Lok® cushion (CIVCO Radiotherapy, Orange City, IA, USA). Patients were positioned supine with both arms placed on the chest. The cushion was molded to cover the region from the lumbar area through the pelvis to the heels, supporting the thighs, knees and lower legs to maintain the lower extremities in an extended position. This immobilization approach was not intended to provide rigid fixation but rather to ensure setup reproducibility while maintaining patient comfort. Therefore, small positional variations of the pelvis and lower extremities were expected and regarded as acceptable for the purposes of this study.

The CT scan parameters included: tube voltage of 120 kVp, automatic tube current modulation, a slice thickness of 1.0 mm, a pitch of 0.5 and a field of view of 500 mm. Images were reconstructed with a soft-tissue kernel on a 512 × 512 matrix.

**Fig. 1:**
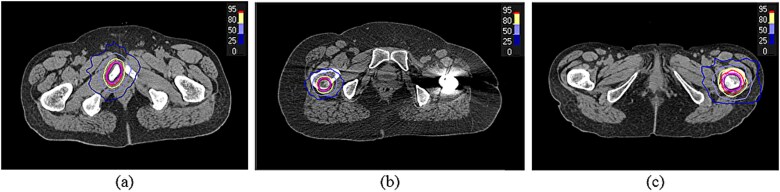
Representative axial views of target volumes and dose distributions. (**a**) Patient L: pubic bone tumor, 32.5 Gy in five fractions; (**b**) Patient U: right femoral tumor, 35 Gy in five fractions; (**c**) Patient W: left femoral tumor, 35 Gy in five fractions. The clinical target volume (CTV) and planning target volume (PTV) are shown. Isodose lines representing 95%, 80%, 50%, and 25% of the prescribed dose are displayed.

In the treatment planning process, a radiation oncologist delineated gross tumor volume (GTV), clinical target volume (CTV) and planning target volume (PTV) based on pathological and radiological findings. A 3-mm margin was added from the GTV to the CTV, and an additional 2-mm margin from the CTV to the PTV. Treatment planning was conducted for SRT. The Monte Carlo algorithm was used to accurately perform dose calculations. The radiation oncologist determined the prescribed dose and number of fractions (8–35 Gy in 1–7 fractions) based on each patient’s clinical condition, tumor location and treatment intent. The prescription dose was defined at the 80% isodose line. A summary of the prescribed doses and fractionation schedules for each group is provided in Supplementary Table S1. Figure 1 shows representative dose distributions for cases using the pubic bone, right femur and left femur as alignment centers.

**Table 1:** Mean values, standard deviations, 95% confidence intervals and sample sizes for positional deviations across six degrees of freedom, categorized based on image registration site

Image registration site	Axis	Mean	SD	95% CI	Number of samples
Spine-based registration group	LR (mm)	−0.09	0.38	−0.11 to −0.06	832
	CC (mm)	0.14	0.43	0.10 to 0.17	
	AP (mm)	0.01	0.31	−0.01 to 0.03	
	Roll (°)	−0.02	0.23	−0.04 to −0.01	
	Pitch (°)	−0.04	0.25	−0.06 to −0.02	
	Yaw (°)	−0.05	0.24	−0.07 to −0.04	
Pubis	LR (mm)	−0.26	0.33	−0.29 to −0.23	445
	CC (mm)	0.04	0.27	0.01 to 0.07	
	AP (mm)	0.4	0.32	0.37 to 0.43	
	Roll (°)	−0.03	0.11	−0.04 to −0.02	
	Pitch (°)	0.05	0.24	0.02 to 0.07	
	Yaw (°)	0.02	0.25	0.00 to 0.04	
Right femur	LR (mm)	−0.15	0.37	−0.19 to −0.11	348
	CC (mm)	−0.2	0.38	−0.24 to −0.16	
	AP (mm)	0.3	0.37	0.26 to 0.34	
	Roll (°)	−0.02	0.31	−0.05 to 0.01	
	Pitch (°)	0.06	0.28	0.03 to 0.09	
	Yaw (°)	0.01	0.20	−0.01 to 0.03	
Left femur	LR (mm)	−0.01	0.30	−0.04 to 0.03	239
	CC (mm)	0.1	0.38	0.06 to 0.15	
	AP (mm)	0.4	0.33	0.36 to 0.44	
	Roll (°)	0.06	0.18	0.04 to 0.08	
	Pitch (°)	0.04	0.18	0.02 to 0.06	
	Yaw (°)	0.00	0.24	−0.03 to 0.03	

Translational deviations are reported in millimeters (mm) along the left–right (LR), anterior–posterior (AP) and cranial–caudal (CC) axes, whereas rotational deviations (pitch, roll, yaw) are reported in degrees (°).

Intra-treatment imaging was performed every 50 s. However, in consideration of the patient’s condition, the imaging interval was occasionally extended to reduce the overall treatment time. As previously described, real-time corrections based on the image registration results were performed using the robotic arm. Rossi *et al*. reported that positional shifts due to patient motion may occur as treatment time elapses, while residual errors remain within submillimeter and sub-degree ranges when robotic tracking compensation is functioning properly [10]. Furthermore, the German Society for Medical Physics consensus by Schmitt *et al*. recommends that if deviations from the planning assumptions are detected by repeated or continuous target-volume localization and are not covered by the applied safety margins, patient or beam repositioning and, if necessary, plan adaptation should be triggered [11]. At our institution, particular emphasis is placed on minimizing residual errors and maintaining consistency with the initial setup position. Therefore, when image registration indicates a deviation exceeding 1 mm or 1°, corrective adjustments are reperformed using the treatment couch rather than relying solely on robotic tracking. These thresholds were established to ensure positional reproducibility within our clinical workflow.

**Fig. 2:**
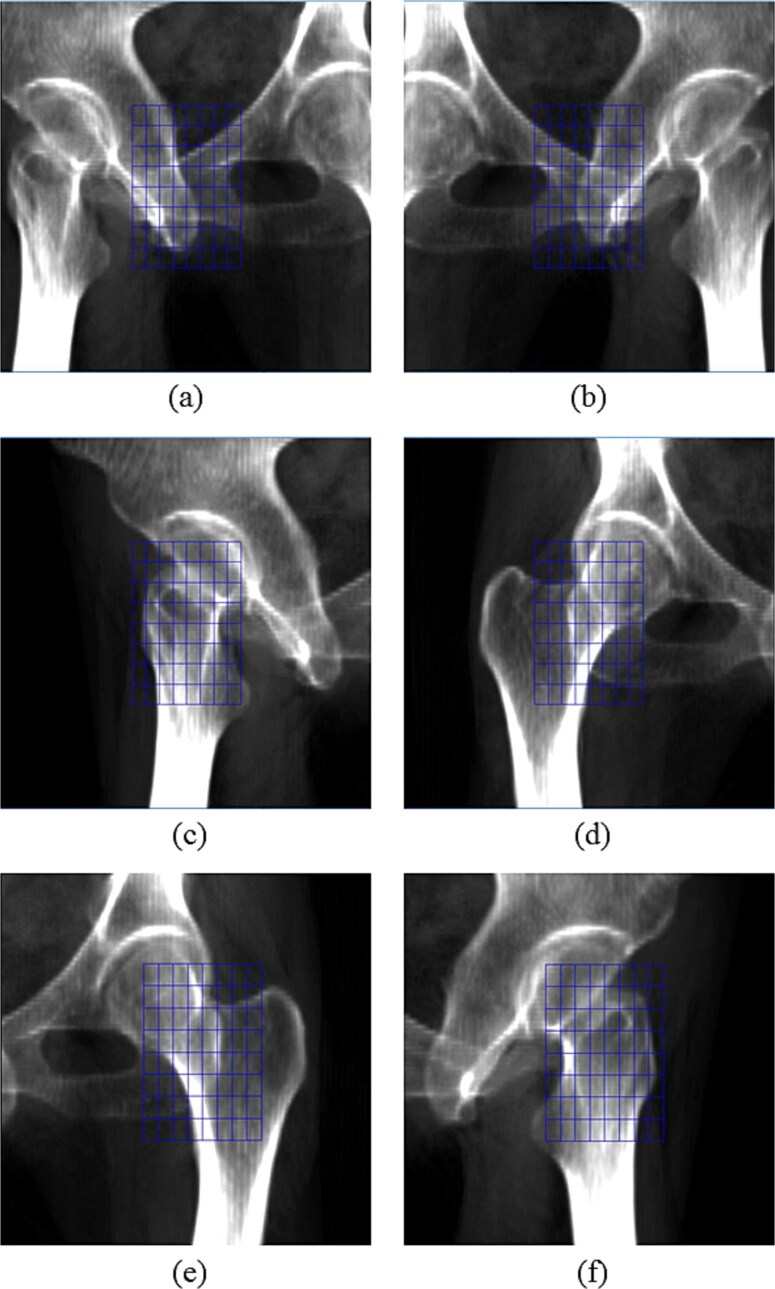
Illustration of regions of interest (ROIs) used for image registration: (**a–b**) pubis, (**c–d**) right femur and (**e–f**) left femur. Each ROI was centered near the target bone and adjusted to include surrounding anatomical landmarks for accurate alignment.

In this study, image registration was performed using the pubis or femoral neck near the irradiation site as the alignment reference, rather than the conventional vertebral body. Figure 2 illustrates the definition of the region of interest (ROI) used for image registration. The ROI size was set to 6 cm in the craniocaudal direction and 4 cm in the left–right direction, corresponding to the default dimensions used in the Xsight Spine Tracking System. The placement of the ROI was guided by the principle of including distinctive bony structures within the registration area whenever possible. This approach is similar to that used in head treatments, where characteristic shapes such as the paranasal sinuses are incorporated into the registration ROI to enhance the robustness of the alignment.

For pubic lesions, the ROI was set to adequately include the edges of the surrounding bony structures. The ROI center was placed near the center of the pubic symphysis. The craniocaudal extent included the superior and inferior borders of the symphysis, whereas the lateral extent encompassed the edges of the obturator foramen and the pubic body. For femoral lesions, the ROI was positioned to include the complex geometry around the proximal femur. This was based on the consideration that, due to the geometry of the femur, rotational changes in the roll direction become more difficult to detect toward the caudal side. The ROI center was placed near the center of the femoral neck. The craniocaudal extent included the superior margin of the femoral head and the region around the ischial tuberosity, whereas the lateral extent included the greater trochanter and the edges of the ischium and the obturator foramen.

To evaluate the effectiveness of the proposed image registration method, all image registration results acquired during the treatment period were grouped according to treatment site, and the positional deviations were analyzed. We calculated the mean and standard deviation for the 6DoF translational axes, left–right (LR), anterior–posterior (AP) and cranial–caudal (CC), as well as for the rotational axes, pitch, roll and yaw, based on anatomical region. To further quantify the accuracy of the measurements, 95% confidence intervals (CIs) were calculated for each mean deviation. The CIs were calculated based on the assumption of a normal distribution. Box plots were generated to illustrate the distribution of positional deviations in each axis and to identify potential outliers. No data transformation or smoothing was applied. These plots are based on all position-corrected data collected in the treatment of all patients, and the data are aggregated by axis.

Furthermore, we quantified the frequency of corrections triggered by deviations exceeding the predefined thresholds. For each patient, the number of corrections per fraction was calculated, and the mean across all fractions was obtained. These patient-level means were then aggregated to calculate the group-level mean and standard deviation. This approach was chosen to avoid bias arising from differences in the total number of fractions among patients.

In addition, for intergroup comparisons, the Spine group was used as the reference group, and positional deviations were compared with those of other treatment sites using two-sided Welch’s *t*-test, which does not assume equal variances. A *P*-value of <0.05 was considered statistically significant. Effect sizes were calculated using Cohen’s *d* to quantify the standardized magnitude of differences relative to the Spine group.

## RESULTS

Representative registrations of DRRs and X-ray images during treatment of pubic, right femoral and left femoral lesions are shown in Supplementary Fig. S1. Image alignment was confirmed by radiation therapists, medical physicists and radiation oncologists based on visual assessment of the consistency between DRRs and X-ray images and evaluation of alignment parameters. In all cases, the alignments were clinically acceptable at the time of treatment.

Table 1 summarizes the mean, standard deviation, CIs and number of samples for translational and rotational positional deviations observed after the initial image registration and during treatment, categorized by tumor location. For both the conventional vertebral body–based registration method used as a reference and the nonspinal bony landmark–based image registration technique evaluated in this study, all mean translational and rotational deviations remained within the predefined institutional clinical tolerance of 1 mm and 1°, respectively. In addition, the 95% CIs for all parameters confirmed that the estimated mean values were statistically stable.

**Fig. 3:**
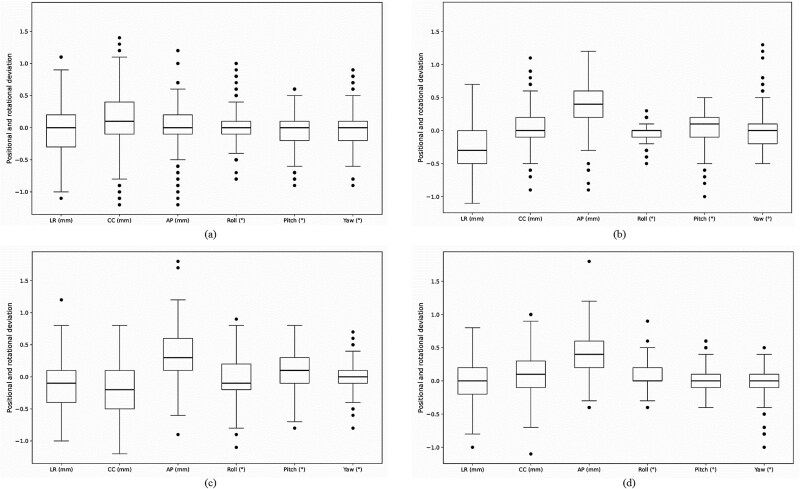
Box plots of positional deviations in six degrees of freedom for (**a**) the spine-based registration group, (**b**) pubis, (**c**) right femur and (**d**) left femur. Translational deviations are presented in millimeters (mm) along the left–right (LR), anterior–posterior (AP) and cranial–caudal (CC) axes, whereas rotational deviations (pitch, roll, yaw) are given in degrees (°). Black circles represent outliers. Each plot displays the median (horizontal line), interquartile range (box) and outliers, defined as values exceeding 1.5 times the interquartile range and marked with black circles.

Figure 3 presents box plots of intrafractional positional deviations across 6DoF (LR, CC, AP, pitch, roll and yaw) for each treatment site. The median values of each group were generally located near zero, and no substantial differences in the overall spread of the distributions were observed among the treatment sites. However, slight differences in the median positions were observed between groups. In particular, in the AP direction, although the magnitude of the deviation was small, the non-spine–based groups showed median shifts in the same direction relative to the spine-based group. Despite overall variability remaining within clinically acceptable limits, potential outliers exceeding the clinical tolerance of 1 mm and 1° were observed in all treatment sites.

The frequency of intrafraction corrections per patient, along with their mean and standard deviation, is shown in Supplementary Table S2. Based on these results, the group-level means (SDs) were 1.1 (1.4) for the conventional spine-based registration, 1.3 (2.3) for the pubic lesion group, 0.6 (0.3) for the right femoral lesion group and 0.9 (0.3) for the left femoral lesion group.

The results of the Welch’s *t*-tests and Cohen’s *d* analyses are summarized in Table 2. Welch’s *t*-tests indicated statistically significant differences across all axes, except for the roll-direction comparison between the spine-based and pubis-based registration groups and that between the spine-based and right femur–based registration groups. In contrast, effect size estimates based on Cohen’s *d* were generally small (|*d*| < 0.5) for most comparisons. Among the 18 comparison items, four showed moderate or larger effect sizes, observed in the CC direction between the spine-based and right femur–based registration groups, and in the AP direction between the spine-based registration group and the other treatment sites.

**Table 2:** Results of Welch’s *t*-tests and effect size analysis for positional deviations relative to the Spine group (two-sided test, α = 0.05)

Axis	Comparison	*t* value	*P*-value	Cohen’s *d*
LR (mm)	Spine vs pubis	8.226	5.85 × 10^−16^	0.473
LR (mm)	Spine vs R femur	2.491	1.30 × 10^−2^	0.158
LR (mm)	Spine vs L femur	−3.527	4.61 × 10^−4^	−0.242
CC (mm)	Spine vs pubis	4.928	9.46 × 10^−7^	0.270
CC (mm)	Spine vs R femur	13.495	2.93 × 10^−37^	0.838
CC (mm)	Spine vs L femur	1.192	2.34 × 10^−1^	0.085
AP (mm)	Spine vs pubis	−20.888	<1 × 10^−78^	−1.235
AP (mm)	Spine vs R femur	−12.887	<1 × 10^−32^	−0.856
AP (mm)	Spine vs L femur	−16.353	<1 × 10^−44^	−1.226
Roll (°)	Spine vs pubis	0.598	0.55	0.032
Roll (°)	Spine vs R femur	−0.343	0.73	−0.023
Roll (°)	Spine vs L femur	−5.917	6.17 × 10^−9^	−0.403
Pitch (°)	Spine vs pubis	−5.854	6.68 × 10^−9^	−0.343
Pitch (°)	Spine vs R femur	−5.869	7.40 × 10^−9^	−0.386
Pitch (°)	Spine vs L femur	−5.331	1.47 × 10^−7^	−0.359
Yaw (°)	Spine vs pubis	−5.280	1.62 × 10^−7^	−0.311
Yaw (°)	Spine vs R femur	−4.695	3.17 × 10^−6^	−0.290
Yaw (°)	Spine vs L femur	−3.116	1.97 × 10^−3^	−0.228

## DISCUSSION

This work represents an initial attempt to assess the feasibility of image registration based on nonspinal bony landmarks, including the pubic bone and femoral neck.

Although the Xsight Spine Tracking System was originally designed for vertebral structures, we confirmed within the treatment-planning system that it could be applied to nonspinal bony anatomy without any software modifications, and that the system functioned properly without errors or functional limitations. The current ROI configuration has functioned effectively in clinical practice, and no cases of registration failure have been encountered to date. However, as the number of treated cases increases, issues that have not yet been observed may arise.

As a practical consideration, the accuracy of rotational direction recognition warrants attention. For the pubic bone, DRRs are highly symmetrical, making rotational misalignments relatively easy to detect even if an angular deviation is present at the initial patient setup. In contrast, as shown in Fig. 1, the femur exhibits pronounced left–right asymmetry in the DRRs due to its anatomical structure. This asymmetry may reduce the detectability of deviations in rotational angle and direction during visual assessment of image registration results. Therefore, accurate patient positioning at the initial setup is considered important for achieving reliable image registration.

Additionally, when the rotational deviation is large during image registration, portions of the pelvic bones may unintentionally appear in the X-ray images. In such cases, even if the numerical registration metrics appear satisfactory, the actual alignment may still be incorrect, as the intensity-based registration algorithm may be influenced by high-contrast pelvic structures outside the intended target, underscoring the importance of visual verification.

Furthermore, although a standardized ROI size was used in this study, variations in patient anatomy or body size may require adaptive adjustment during treatment to prevent unintended inclusion of surrounding pelvic bones. These insights may serve as practical guidance in clinical implementation.

As shown in Fig. 3, the distributions of intrafractional positional deviations during treatment were generally small across all groups. Translational deviations were mostly within ~±0.5 mm, and rotational deviations were within ~±0.5°, indicating that patient positioning was maintained within clinically acceptable limits. The boxplots demonstrate substantial overlap among groups in all translational and rotational directions, with similar medians and interquartile ranges, suggesting comparable overall positioning performance regardless of the reference structure used for image registration.

Nevertheless, outliers exceeding the predefined clinical tolerance of 1 mm and 1° were observed. These deviations either gradually increased over the course of treatment or abruptly increased compared with the preceding measurements, suggesting that they were primarily caused by patient motion. The mean number of re-registrations showed little variation across cases. However, as shown in Supplementary Table S2, two patients treated for thoracic bone metastases and one patient treated for pubic bone metastasis required more than 10 corrections. This was likely attributable to the difficulty of maintaining the same posture for a prolonged period, given that the treatment sites involved painful bone metastases. In these patients, the administration of analgesics reduced the number of re-registrations during treatment.

As shown in Table 2, Welch’s *t*-test demonstrated statistically significant differences in all comparisons except for the roll direction between the spine-based group and the pubic bone–based group, and between the spine-based group and the right femoral-based group. One possible reason for this finding is the high statistical power of the analysis, as multiple fractions from each patient were included, resulting in a large effective number of observations. Under such conditions, even small differences in mean values are more likely to be identified as statistically significant. In addition, positional deviations were analyzed as signed values rather than absolute values in this study. Consequently, the statistical comparisons may have emphasized differences in mean values that included directional components across treatment sites.

Effect size analysis using Cohen’s *d* revealed moderate or greater effect sizes in 4 out of 18 comparisons: in the AP direction between the spine-based group and both the pubic bone–based and femoral-based groups, and in the CC direction between the spine-based group and the right femoral-based group. These results likely reflect the fact that the standard deviations were comparable between the spine-based and comparison groups, while the differences in mean values including directional components were relatively large, leading to increased standardized effect sizes. This tendency is consistent with the distributions shown in the box plots in Fig. 3. Furthermore, the CC and AP directions, in which moderate or greater effect sizes were observed, correspond to directions in which the patient’s trunk is generally stabilized by immobilization devices, yet involuntary or subtle patient motion may still occur during treatment. Such motion may have contributed to the observed intergroup differences. In contrast, in the LR direction, positional deviations were less likely to occur due to the stepped structure of the immobilization device designed to conform to the patient’s body shape, which may explain the smaller intergroup differences and effect sizes observed.

Nevertheless, in all directions, the absolute values of the mean positional deviations were small, remaining below 0.5 mm. The box plots and 95% CIs also indicated no differences that would be considered clinically problematic. Although statistically significant intergroup differences were detected, the absolute magnitude of the mean differences was small and did not reach a level that would affect treatment accuracy. Therefore, the statistically significant differences and effect sizes observed in this study are likely to primarily reflect measurement precision and characteristics of the data distribution. Although their clinical impact appears limited, these findings should be interpreted with caution, and further investigation may help clarify their potential clinical relevance.

Stable patient positioning is an essential prerequisite for the feasibility and effectiveness of our proposed approach, in which the target itself serves as the alignment center. At our institution, fixation devices are utilized not to enforce rigid immobilization but to support stable body positioning. Therefore, movements in the lumbar or pelvic region during treatment may have resulted in displacement of the bony anatomy. The use of and the effectiveness of fixation devices in reducing setup uncertainties have been reported by Udayashankar *et al*. and Janssen *et al*. [12, 13]. Consequently, additional strategies to further minimize patient motion should be considered in our future clinical implementation.

This study has several limitations. First, the number of cases was relatively small (*n* = 24), and therefore the present analysis should be regarded as an exploratory investigation providing a preliminary assessment of feasibility. Larger studies with expanded patient cohorts are required to validate and generalize the findings. Second, the proposed method does not explicitly account for anatomical deformation of the tumor or surrounding tissues that may occur during the treatment course, and patient-specific factors such as posture or body habitus may further interfere with accurate detection of bony structures, potentially affecting image registration accuracy. A major limitation of this study is the lack of independent validation using an anthropomorphic pelvic phantom, primarily due to the current unavailability of a suitable phantom that reproduces complex pelvic anatomy for end-to-end testing. As a result, the tracking algorithm was evaluated based on its own registration outputs, and visual inspection alone cannot confirm true geometric accuracy relative to a known ground truth. Although the present results demonstrate the clinical feasibility of the proposed approach, phantom-based validation remains an important next step. Future studies will therefore focus on validating ROI settings using appropriate phantoms and conducting clinical evaluations in larger patient cohorts to further confirm the robustness of this method.

Despite this study’s limitations and potential areas for investigation, the proposed method demonstrates the feasibility of image registration using an alignment center outside the vertebral body by leveraging existing system capabilities. This approach may offer a practical alternative in clinical settings where image registration is typically performed using bony anatomy that lies far from the target. By allowing direct alignment based on the lesion itself, it may improve positional accuracy in the region surrounding the tumor.

## Supplementary Material

rrag028_Supplementary
