# Supplementary figures and images for: Feasibility of nonspinal bony landmark-based image registration in CyberKnife ® robotic radiotherapy for pelvic and femoral bone tumors
Source: J Radiat Res. 2026 Apr 24;67(3):473–80. doi: 10.1093/jrr/rrag028 (PMC13202333; doi:10.1093/jrr/rrag028)

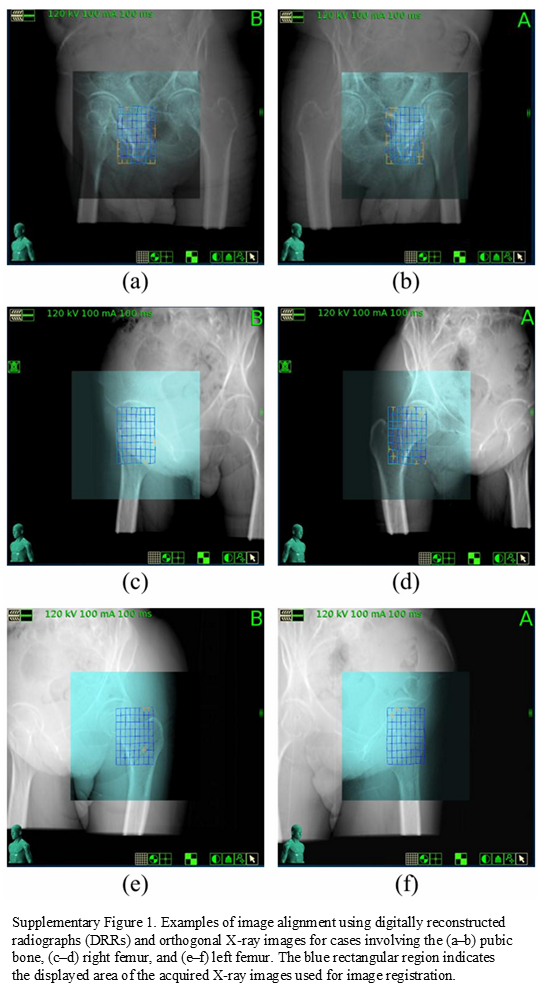

Supplement: rrag028_Supplementary [file rrag028_supplementary.zip › Supplementary_Figure_1_R1_rrag028.tif]
